# Morphology of the immune cells in the wall of the human uterine tube and their possible impact on reproduction—uterine tube as a possible immune privileged organ

**DOI:** 10.3389/fcell.2024.1325565

**Published:** 2024-03-07

**Authors:** Kristína Visnyaiová, Ivan Varga, Claudia Feitscherová, Lada Pavlíková, Jozef Záhumenský, Renáta Mikušová

**Affiliations:** ^1^ Second Department of Gynecology and Obstetrics, Faculty of Medicine, Comenius University in Bratislava and University Hospital, Bratislava, Slovakia; ^2^ Institute of Histology and Embryology, Faculty of Medicine, Comenius University in Bratislava, Bratislava, Slovakia; ^3^ Department of Rehabilitation Studies, Faculty of Health Care Studies, University of Western Bohemia, Pilsen, Czechia

**Keywords:** uterine tube, macrophage, dendritic cell, T lymphocyte, B lymphocyte, natural killer cell, mast cell, fertility

## Abstract

The uterine tube, as well as other parts of the upper female reproductive system, is immunologically unique in its requirements for tolerance to allogenic sperm and semi-allogenic embryos, yet responds to an array of sexually transmitted pathogens. To understand this dichotomy, there is a need to understand the functional morphology of immune cells in the wall of the uterine tube. Thus, we reviewed scientific literature regarding immune cells and the human uterine tube by using the scientific databases. The human uterine tube has a diverse population of immunocompetent cells representing both the innate and adaptive immune systems. We describe in detail the possible roles of cells of the mononuclear phagocyte system (macrophages and dendritic cells), T and B lymphocytes, natural killer cells, neutrophils and mast cells in association with the reproductive functions of uterine tubes. We are also discussing about the possible “immune privilege” of the uterine tube, as another mechanism to tolerate sperm and embryo without eliciting an inflammatory immune response. In uterine tube is not present an anatomical blood-tissue barrier between antigens and circulation. However, the immune cells of the uterine tube probably represent a type of “immunological barrier,” which probably includes the uterine tube among the immunologically privileged organs. Understanding how immune cells in the female reproductive tract play roles in reproduction is essential to understand not only the mechanisms of gamete transport and fertilization as well as embryo transport through the uterine tube, but also in improving results from assisted reproduction.

## 1 Introduction

Uterine tubes (fallopian tubes, oviducts) are important organs of the female internal reproductive system, responsible for cardinal processes needed for successful reproduction. The uterine tube is the only tubular organ of the human body that, even under physiological conditions, performs a transport function in opposing directions. The picked-up oocyte released during ovulation or formation of the early embryo is transported proximally, to the uterine cavity. Concomitantly, the uterine tube also facilitates transport of spermatozoa distally, to the ampullary portion, where fertilization usually occurs. However, the uterine tube has many other unique functions, such as sperm selection (an essential mechanism for preventing polyspermatic fertilization) or formation of a unique tubal fluid. Tubal fluid is important not only during fertilization but activates sperm before fertilization and nourishes the embryo during transport to the uterine cavity (Csöbönyeiová et al., 2022b; Varga et al., 2022; Hamranová et al., 2023).

The uterine tube, as well as other parts of the upper female reproductive system, is immunologically unique in its requirements for tolerance to allogenic sperm and semi-allogenic embryos, yet responds to an array of sexually transmitted pathogens. Although uterine tubes are considered sterile, microorganisms can ascend from the lower reproductive tract, causing pelvic inflammatory disease and/or reproductive alterations such as ectopic pregnancy (Caven and Carabeo, 2023; Jenabi et al., 2023; Pant et al., 2023) and tubal infertility (Hoenderboom et al., 2019; Liu et al., 2022). In general, uterine tube diseases account for 25%–35% of female factor infertility. Pelvic inflammatory disease is the most common cause of tubal disease, representing more than 50% of cases (Chua et al., 2017). However, the lumen of uterine tubes is not sterile. Uterine tubes harbor highly variable microbial communities among women, especially a variety of bacteria growing in mildly alkaline conditions. These diverse microbial communities of uterine tubes are affected by hormones and antibiotics, and exhibit biogeographical tropism (Liptáková et al., 2022). Epithelial cell secretions filled with several cytokines and chemokines from the human female upper reproductive tract inhibit sexually transmitted pathogens (including *Neisseria gonorrhoeae* and yeast *Candida albicans*), as well as reduce HIV-1 infection of target cells. In contrast, these secretions have no inhibitory effect on *Lactobacillus* (Wira et al., 2011). Probably because of this specific tubal microenvironment, the presence of lymphatic follicles in the wall of uterine tubes (indicating a massive presence of B lymphocytes and their activated antibody-producing plasma cells, usually present in the mucosae of other organs) is rare yet detectable during histological examination only in 2.1% of surgically removed healthy uterine tubes (Hunt and Lynn, 2002).

There is a diverse range of immunocompetent cells in uterine tubes. Ulrich et al. (2022) in their study applied single-cell RNA-sequencing to analyze more than 59.000 unselected cells from 10 specimen of human uterine tubes from 4 healthy subjects. They define 12 major cell types in healthy uterine tubes, from 4 are immune cells (B lymphocytes, T lymphocytes/NK cells, mast cells and macrophages). Ramraj et al. (2018), in a flow cytometric analysis of immunocompetent cells from dispersed tubal specimens, indicates that the predominant cell type is M1 macrophages (20% of total cells); followed by regulatory T lymphocytes (7%), CD8 cytotoxic T lymphocytes (3%), M2 macrophages (1%), perforin-positive natural killer (NK) cells (0.52%), and granzyme B-positive NK cells (0.13%). The aim of this narrative review is to present a comprehensive overview of the immune cell populations in the wall of human uterine tubes. We describe in detail the possible roles of cells of the mononuclear phagocyte system (macrophages and dendritic cells), T and B lymphocytes, NK cells, and mast cells in association with the reproductive functions of uterine tubes. All mentioned immunocompetent cells residing in the reproductive tract play paradoxical roles since they maintain immunity against pathogens yet also establish immune tolerance for sperm and embryos/fetuses (Lee et al., 2015). However, we are also discussing about the possible mechanism of “immune privilege” within the lumen of the uterine tube, as another mechanism to tolerate the foreign antigens as sperm and embryo without eliciting an inflammatory immune response. Understanding how immune cells in the female reproductive tract play roles in reproduction is essential to understand not only the mechanisms of gametes transport and fertilization as well as embryo transport through the uterine tube, but also in improving results from assisted reproduction and clinical embryology.

## 2 Methods

Recent and relevant scientific literature was reviewed herein on the topic of the functional morphology of immune cells in the wall of the human uterine tube. Scientific literature regarding morphologically different types of immune cells and the human uterine tube was reviewed by using scientific databases PubMed/Medline, SCOPUS, and Web of Knowledge.

A total of 109 references consisting of reviews, case reports, and original articles from the fields of histology, immunology, gynecology, and reproductive medicine published mostly in recent years were selected to document the present manuscript. The collected data were organized as a narrative literature review synthesis structured in paragraphs.

## 3 Cells of the mononuclear phagocyte system in the uterine tube

The mononuclear phagocyte system is defined as a cell lineage in which committed bone marrow progenitors give rise to monocytes of the peripheral blood and tissue macrophages. This nomenclature and definition were first time proposed by a Dutch scientist Ralph van Furth and his co-workers in 1972 (van Furth et al., 1972). In the following years, dendritic cells were also incorporated into this system. The mononuclear phagocyte system represents a critical regulator of innate and adaptive immune responses. Additionally, tissue macrophages also play a crucial role in tissue homeostasis, wound healing, and tissue regeneration in prenatal as well as postnatal development (Hume et al., 2019; Sreejit et al., 2020; Miah et al., 2021). Macrophages are a major cell population in most of the tissues in the body; and their numbers increase further in inflammation, wounding, and malignancy (Hume, 2006). There are two hypotheses about the exact origin of tissue macrophages. First, tissue-resident macrophages are a separate lineage seeded exclusively during embryonic development and maintained through adulthood by longevity and self-renewal. Second, other experiments suggesting that during postnatal life, circulating blood monocytes can replace tissue resident macrophages in all major organs and adopt their tissue-specific gene expression (Varol et al., 2015; Hume et al., 2019). In uterine tubes, macrophages and their pro-inflammatory and anti-inflammatory functions play a critical role during *Chlamydia* infection and subsequent development of hydrosalpinx and/or pelvic inflammatory disease and ectopic pregnancy (Harvie et al., 2019; Wang et al., 2020; Zhang et al., 2022).

### 3.1 Macrophages in the uterine tube

In uterine tubes, macrophages are localized within the epithelium and in the connective tissue of the lamina propria. In an immunohistochemical study by Gaytán et al. (2007), performed on uterine tubes in different phases of the menstrual cycle, macrophages exhibited cyclic changes in both numbers and location in the uterine tube mucosa. Macrophages were relatively abundant in both the epithelium and the underlying connective tissue (lamina propria) during the early follicular phase, yet were scarce—particularly within the epithelium—during the late follicular phase. During the luteal phase, macrophages exhibited relevant changes in numbers, anatomical location, and morphology. Macrophages were abundant within the epithelium during the early and mid-luteal phases. These macrophages exhibited dendritic features with cytoplasmic projections among epithelial cells, even located at the apical zone of the epithelium. During the late luteal phase, macrophages were still abundant but were preferentially located at the basal zone of the epithelium. Changes in macrophages located in the lamina propria were not as relevant (Gaytán et al., 2007). The number of macrophages decreased with age, in postmenopausal women (Safwat et al., 2008; Rodriguez–Garcia et al., 2021).

Lu et al. (2023) was the first to describe an interesting immune phenomenon: macrophage extracellular trap formation during sperm phagocytosis due to interactions between sperm and macrophages *in vitro* (cell cultures co-incubation). This is a possible mechanism of sperm phagocytosis by macrophages; morphologically abnormal and hypomotile sperm of patients with asthenozoospermia (reduced sperm motility) are more effectively cleared by macrophage extracellular traps compared with sperm from healthy patients. It is possible that such a mechanism of sperm selection also takes place within the uterine tubes. However, this preliminary study by Lu et al. (2023) was realized in cell cultures, and further research will be needed to confirm whether the same mechanism applies *in vivo*, inside the uterine tube. Moreover, spermatozoa interaction with tubal epithelium *in vitro* modifies expression of cytokines, chemokines and growth factors from tubal epithelial cells (Mousavi et al., 2021).

The role of macrophages in the pathogenesis of tubal ectopic pregnancy is not clear, but macrophages might dysregulate both tubal motility and propensity of the uterine tube. In the early or mid-luteal phases, high progesterone levels could recruit macrophage infiltration in the uterine tubes and enhance production of prostaglandin E2 by macrophages. Macrophage type M1 is also capable of secreting large quantities of interleukin 6. Notably, progesterone, prostaglandin E2, and interleukin 6 restrain smooth muscle contraction and induce dysfunction of the ciliated epithelium; resulting in retention of the embryo within the uterine tubes. Macrophages are capable of producing inflammatory cytokines and growth factors (such as interleukin 1 alpha, tumor necrosis factor alpha, and transforming growth factor beta), which could induce a marked upregulation of molecules necessary for embryo implantation such as leukemia inhibitory factor, indicating a crucial role of macrophages in facilitating a tubal environment permissive for embryo implantation (Wang et al., 2019).

Macrophages are abundant in the wall of the uterine tube; however, histiocytic lesions of uterine tubes—the presence of sheets and clusters of histiocytes (an alternative term for tissue resident macrophages in histopathology)—are relatively rare (Tran and Holloway, 2021).

### 3.2 Dendritic cells in the uterine tube

Dendritic cells are specialized antigen-presenting cells; orchestrating innate and adaptive immunity during infections, autoimmune diseases, and malignancies (Kvedaraite and Ginhoux, 2022). After capturing antigens, dendritic cells move to nearby regional lymph nodes via efferent lymph vessels as they mature and then present those antigens to naive T lymphocytes for activation or tolerance induction. There are two main lineages of human dendritic cells: conventional (types 1 and 2) and plasmacytoid. Different subtypes of conventional dendritic cells have different functions: some have an anti-inflammatory function, whereas others produce pro-inflammatory cytokines tumor necrosis factor alpha and interleukin 6. Plasmacytoid dendritic cells were initially called interferon-producing cells because they rapidly produce large quantities of type I interferons after recognizing nucleic acids of viruses or bacteria (Ohteki et al., 2021). Plasmacytoid dendritic cells also induce tolerance through interferon alpha production, by suppressing T lymphocyte proliferation and inducing regulatory T lymphocytes. By using flow cytometry, Shaw et al. (2011) found a relatively large percentage of CD123+ plasmacytoid dendritic cells in human uterine tube and a significantly higher percentage of CD123+ plasmocytoid dendritic cells compared with CD11c+ conventional dendritic cells. Given that the uterine tube is exposed to allogeneic spermatozoa and the semi-allogeneic embryo, and must exhibit tolerance toward these cells for reproduction, plasmacytoid dendritic cells might play a role in facilitating a tolerant phenotype within the tubal microenvironment (Shaw et al., 2011).

### 3.3 Langerhans cells in the uterine tube

Langerhans cells are specific antigen-presenting dendritic cells localized typically in the epidermis; however, they are present in other surface and lining epithelial tissues of the human body, as in the vagina and uterine cervix. Hagiwara et al. (1998) investigated human uterine tubes by electron microscopy and immunohistochemistry by using anti-CD1a antibodies. They discovered Langerhans cells in the tubal epithelium. Moreover, Rabi et al. (2014) described various subtypes of Langerhans cells in the human uterine tube. In accordance with their immunohistochemical study, CD1a-positive Langerhans cells were significantly fewer and smaller in diameter than zinc iodide–osmium-positive Langerhans cells in the uterine tube and both types of cells were significantly more prevalent in *postpartum* tubes. They described perivascular clusters of zinc iodide–osmium-positive Langerhans cells in tissues from the *postpartum* tube, as well as close association of CD1a-positive Langerhans cells with CD4^+^ and CD8^+^ T lymphocytes in the *postpartum* uterine tube. The role of Langerhans cells in the upper female reproductive tract is not fully understood. Tubal Langerhans cells have typical morphological features of Langerhans cells localized in other epithelia, as extended cytoplasmic projections along the basal part of the epithelium and presence of intracytoplasmatic rod-shaped Birbeck granules (visible only by electron microscopy) (Hagiwara et al., 1998).

## 4 T lymphocytes in the wall of the uterine tube

T lymphocytes represent the dominant immune cell population in the healthy uterine tube and account for approximately 40%–60% of all leucocytes. They are predominantly localized as single cells within the epithelium, along the basement membrane. Focused on the epithelium of the uterine tube, intraepithelial T lymphocytes represent the dominant lymphoid subset of human tubal epithelium. In the tubal epithelium, the average ratio of CD3^+^ T lymphocytes to epithelial cells is 1:16. Intraepithelial B lymphocytes are 4× less frequent. The average ratio of immune cells to epithelial cells is 1:400 for CD4^+^ T lymphocytes and 1:15 for CD8^+^ T lymphocytes (Ardighieri et al., 2014; Rigby et al., 2022). Most intraepithelial lymphocytes express estrogen receptor beta at both mRNA and protein levels (Ulziibat et al., 2006).

Surprisingly, for many decades tubal intraepithelial T lymphocytes were described as tubal “basal cells” in various morphological publications; including in official histological nomenclature, the *Terminologia Histologica* (FICAT, 2008). These tubal “basal cells” exhibit small, hyperchromatic nuclei and pale cytoplasm (clear cytoplasmic halo). They are located in the epithelium adjacent to the basement membrane, and are non-mitotically active. Varga et al. (2019) conducted an immunohistochemical study that confirmed that these tubal “basal cells” are intraepithelial T lymphocytes; mostly regulatory T lymphocytes (CD3^+^, CD8^+^, CD45RO^+^, CD4^−^, CD20^−^, CD56^−^, and granzyme B^−^). Intraepithelial T lymphocytes can be involved in immune tolerance, which could lead to the tolerance of non-self cells (sperm) and partially non-self cells (a developing embryo) without activating local immune responses. Considering the important role of regulatory T lymphocytes in modulating immunological balance and supporting the embryo, further research is required on the distribution patterns and functions of regulatory T lymphocytes in the uterine tubes, especially regarding women with tubal ectopic pregnancy.

## 5 B lymphocytes and lymphoid follicles in the uterine tube

B lymphocytes account for approximately 5%–10% of all leucocytes in healthy uterine tubes. They are less frequently detected than T lymphocytes and predominantly localize within the connective tissue lamina propria (Rigby et al., 2022). Only a small number of B lymphocytes as well as B dependent lymphoid follicles are present in the mucosa of the uterine tube (Wang et al., 2019; Mills, 2020). In the histological study of Hunt and Lynn (2002), lymphoid follicles within the tubal mucosal folds were present only in 2.1% of bioptic samples. Even the mucosae of most of other tubular organs have a higher prevalence of lymphoid follicles; the cited authors consider their occurrence as evidence of tubal mucosa associated lymphoid tissue as a potential protective mechanism.

Wollen et al. (1994) histologically studied sections from the human uterine tubes of women who were using an intrauterine contraceptive device. The number of cells positive for immunoglobulins IgA, IgG, or IgM (activated forms of B lymphocytes) was significantly higher in the intrauterine contraceptive device user group than in the control group. Therefore, the intrauterine contraceptive device can incite inflammatory cells, resulting in inflammation of the uterine tube; and thus might interfere with the immunological function of the uterine tube and the uterine tube’s role in reproduction.

## 6 NK cells in the wall of the uterine tube

NK cells are relatively common in the mucosa of the uterus (endometrium), especially during pregnancy. They represent 70% of endometrial/decidual leukocytes during pregnancy. These uterine NK cells differ from peripheral blood NK cells (less cytotoxic) and exhibit critical immunomodulatory functions with the potential to control embryo implantation and trophoblast invasion, regulate placental vascular remodeling, and facilitate embryonic/fetal growth (Lapides et al., 2023). In the human uterine tube, NK cells are less numerous and are present in the epithelium, lamina propria, and tubal wall. In the epithelium they are evenly distributed between the epithelial cells with an average ratio of NK cells to epithelial cells of 1:70 (Ardighieri et al., 2014). Shaw et al. (2011) demonstrated the presence of CD56dimCD16^-^ NK cells in the non-pregnant uterine tube. NK cells at the ectopic tubal pregnancy implantation site express lower percentages of perforin and granulysin, but express a higher percentage of tumor necrosis factor related apoptosis inducing ligand) than do ectopic pregnancy uterine decidual and peripheral NK cells (Laskarin et al., 2010).

## 7 Neutrophils in the wall of the uterine tube

In the human body, neutrophils are the most dominant cellular component of innate immunity. As the human body’s first line of defense against microorganisms, neutrophils execute a variety of potent effector mechanisms for mediating innate immunity. In the cervix and vagina, neutrophils contribute to removal of harmful microbes entering the vagina, or phagocytosis of damaged or dead sperm (Pandya and Cohen, 1985; Milligan et al., 2001). Regarding the uterine tube, the role of neutrophils is largely unknown. Uterine tube neutrophils exhibit a phenotype distinct from peripheral blood neutrophils (expressed significantly higher levels of CD64, human class II histocompatibility antigen DR, gamma-interferon, and vascular endothelial growth factor), suggesting functional activation of innate immune defense in the female reproductive tract as well as a potential role in maintaining normal uterine tube physiology (Smith et al., 2006). In animals (e.g., buffalo), neutrophils are present within the epithelium and also inside the lumen of the uterine tube around the estrus period. Additionally, the preovulatory tubal fluid suppresses sperm phagocytosis by neutrophils via prostaglandin E2 action (Marey et al., 2016; Yousef et al., 2019).

## 8 Mast cells in the wall of the uterine tube

Mast cells (mastocytes) are common in connective tissue or in the mucosa of many organs; including the skin, airways, and digestive tract. They are also present in male and female reproductive organs. Mast cells are involved in inflammatory, hypersensitivity, and fibrotic disorders; their effects are hypothesized to be mediated by biogenic amines, proteoglycans, and prostaglandins but also by neutral proteases. They are widely known for their role in allergic reactions via their binding to immunoglobulin E receptor. In addition, there is growing evidence for a role of mast cells in host immune defense and autoimmunity (Weidinger et al., 2003). From a histological point of view, mast cell density is higher in the muscle layer of uterine tube than in tubal lamina propria. Most mast cells of the muscle layer are more closely related to smooth muscle cells than to blood vessels (Sandvei et al., 1986).

Numerous authors have demonstrated expression of estradiol and progesterone receptors in human, mouse, and rat mast cells. For this reason, many mast cell-related pathophysiological alterations have a different prevalence in females and males; e.g., there is much higher asthma prevalence in women at reproductive age compared with that in men, and serum levels of estradiol and progesterone have been directly correlated with the clinical and functional features of asthma (Zierau et al., 2012). Although COVID-19 hyper-inflammation and post-COVID-19 syndrome might be based on mast cell activation syndrome, the available clinical data do not provide grounds for treating this mechanism as significantly increasing the risk of abnormal female reproductive function (Szukiewicz et al., 2022).

The exact role of mast cells in the wall of the uterine tube is largely unknown. Weidinger et al. (2003) demonstrated that the mast cell product tryptase interacts with human spermatozoa during migration through the female genital tract and directly reduces sperm motility. In women who were using an intrauterine contraceptive device, the number of mast cells in the wall of the uterine tube was higher than in a control group of non-pregnant women. This increased number of mast cells in intrauterine contraceptive device users might be a factor in the pathogenesis of pelvic inflammatory disease and the ectopic pregnancies that occur in intrauterine contraceptive device users (Sandvei et al., 1986). From an immunological point of view, mast cells also play an important role in shaping immune responses in *Chlamydia* reproductive tract infection through both effector cell recruitment and modification of the chemokine microenvironment (Mayavannan et al., 2023).

## 9 Possible “immune privilege” of uterine tube

The immune privilege represents a specific tissue microenvironment where the systemic immune responses to allo- and autoantigens are reduced. Immune privilege is thought to reflect an adaptation to protect vital structures from damage by inflammatory responses directed against pathogens. It was originally believed that antigens in immune-privileged sites are separated from the immune system by anatomical barriers (to be sealed from the blood circulation) and therefore ignored. Nowadays we know that immune privilege is maintained thanks to immune cells by an active rather than passive process (Hong and Van Kaer, 1999). Immune privileged organs include the eye (Du and Yan, 2023; Wang et al., 2023) and the brain (Rustenhoven and Kipnis, 2022; González-Hernández and Mukouyama, 2023) as well as the pregnant uterus (Zhang et al., 2016) and testis (Kaur et al., 2021). However, so far no one has described a similar “immune privilege” inside the uterine tube.

Male germ cells are immunogenic. The immune privilege is well described in male genital system; mostly in testis, but less direct evidence support that the whole epididymis is immune-privileged (Mital et al., 2011). The immune microenvironment of the male genital organs, specifically the testis and epididymis, is characterized by the formation of a specific barrier between the immune system and haploid sperm. These barriers are the blood-testis barrier and blood-epididymis barrier. The ultrastructural composition of both mentioned anatomical barriers is well known and consists of Sertoli cell-Sertoli cell tight junctions or tight junctions between the epithelial cells in the epididymal duct (Gregory and Cyr, 2014; Luaces et al., 2023). However, both barriers contain also functional parts, the so-called physiological and immunological barriers (Mital et al., 2011), which are similarly important for the immune privilege. Inside ovary, the presence of blood-follicle barrier is discussed. From anatomical point of view, the layers of this barrier are: the vascular endothelium, endothelial basement membrane, the thecal interstitium, the follicular basement membrane, and the membrana granulosa (Siu and Cheng, 2012). But according to our knowledge, no similar anatomical barrier is inside the uterine tube.

The presence of anatomical blood-tissue barrier is not the only basis for the immune privilege. Similar important is the presence of immunological barrier. E.g., in testis, T lymphocytes play an imperative role in testicular immunity, thus maintaining a tolerance status in terms of the physiological condition and arousing an immune response under threat of infection and tumorigenesis (Gong et al., 2020). From embryological point of view, the male epididymis is analogous to female uterine tube. Inside epididymis, numerous immune cells preventing induction of an autoimmune reaction as, mononuclear phagocytic cells, CD4^+^ T lymphocytes, and CD8^+^ T lymphocytes, which are located throughout the epididymal epithelium and interstitial space (Da Silva and Smith, 2015; Zhao et al., 2022). Similar key cells of the immune system have also been described in the uterine tube. This is also why it is possible that the immune cells of the uterine tube represent a type of “immunological barrier,” which in a certain way includes the uterine tube among the immunologically privileged organs. Additionally, anti-inflammatory cytokines, TGFB1 and IL10, are strongly and constantly expressed in the uterine tube epithelial and endothelial cells of the surrounding vasculature (Yousef et al., 2016). This constant expression of both epithelial-derived T helper type 2-driving (Th2, anti-inflammatory) cytokines suppressed the immune system, thereby inducing a state of immune-tolerance toward sperm and early embryo in the uterine tube. These findings suggest that under physiological conditions, the uterine tube mucosal epithelium provides a strong and stable anti-inflammatory environment (Marey et al., 2020).

To our knowledge, only one study investigated *in vivo* the uterine tube (even only its intramural portion) as an immunoprivileged organ in baboons (the anatomical structure of baboon’s female reproductive system resembles that of human). Hernandez et al. (2021) placed silver nitrate loaded fiber devices in uteruses and intramural portions of uterine tubes of baboons to probe the fibrotic response. Cited authors found no evidence of inflammation, collagen deposition or abnormal immune cell infiltration in either the endometrium or intramural portion of uterine tube. In other hand, the same electrospun fibers initiating fibrosis after subcutaneous application. This experimental study may be the first confirmation that at least the intramural portion of the uterine tube is an immune privileged organ.

## 10 The role of tubal fluid in immune privilege of uterine tube

The tubal fluid inside the uterine tube consists of components that are either passively or actively transported over the tubal epithelial barrier from the circulating blood or the interstitial tissue, and/or secreted by the tubal epithelial cells (Leese et al., 2001). Probably the uterine tube-specific lymphatic lacunae (width lymphatic capillaries), which run through the central part of each uterine mucosal fold and fimbria, may play an important role in the formation and/or absorption of tubal fluid (Varga et al., 2018; Csöbönyeiová et al., 2022a). The composition of tubal fluid differs significantly from the blood plasma (Ménézo et al., 2015). Tubal fluid protein concentrations are low compared to those of blood serum; although some proteins such as albumin are in abundance (Pérez-Cerezales et al., 2018). The unique tubal liquid microenvironment contains a variety of molecules, including tubal extracellular vesicles (both exosomes and microvesicles), specific glycoproteins as oviductin, nutrients such as glucose, lactate, pyruvate and amino acids, and bicarbonate ions. All these components provide an optimal environment for sperm survival and capacitation, fertilization and early embryo development in the uterine tube (Harris et al., 2020; Cajas et al., 2021; Zhao et al., 2022). The volume, chemical composition and even temperature of the tubal fluid is influenced by the cyclically changing level of female sex hormones (Saint-Dizier et al., 2019). Additionally, tubal fluid contains immune cells as neutrophils (Marey et al., 2013), and macrophages, which may arise from peritoneal macrophages that migrate into the uterine tubes (Haney et al., 1983). The significant presence of neutrophils in the tubal fluid was described in the bovine especially during preovulatory period (Marey et al., 2016). Little is known about the tubal fluid local immunological microenvironment and how tubal fluid immune cells interact with sperm and embryo. Insemination always stimulates neutrophil migration into the female reproductive tract, which eliminates excess spermatozoa and bacterial contaminants introduced by the coitus (Alghamdi and Foster, 2005). The composition of tubal fluid/secretion of tubal epithelial cells through mainly prostaglandin E2 suppresses the phagocytic activity of neutrophils for sperm, which is also hormonally regulated through luteinizing hormone (Marey et al., 2013). In the time window of ovulation, follicular and tubal fluids seem to have opposite effects on the uterine tube immunotolerance. The follicular fluid collected from buffalo pre-ovulatory follicles enhanced sperm phagocytosis by neutrophils through the formation of neutrophil extracellular traps and hydrogen peroxide formation, whereas tubal fluid inhibited this activity *in vitro* (Yousef et al., 2016).

The tubal fluid bidirectionally carries signaling molecules between sperm/pre-implantation embryo on the one hand and tubal epithelial cells and immune cells on the other hand. Arrival of spermatozoa and/or preimplantation embryo within the uterine tube is now known to regulate gene expression in tubal epithelial cells, inducing up- and downregulation of various proteins. The sperm-uterine tube interaction generates an anti-inflammatory immune response, which supports sperm survival until fertilization (Yousef et al., 2016). Moreover, tubal fluid appears to protect against activated leucocyte-induced sperm DNA fragmentation, thus preserving the integrity of the paternal genome (Navarrete Gómez et al., 2009). The positive influence of the tubal fluid on early embryonic growth and development is well established in non-human species (Rizos et al., 2016). During the embryo-uterine tube interaction, the embryo might play a role as a modulator of the immune system in the maternal tract, inducing the downregulation of immune related genes to allow the refractory uterine tube and uterus to tolerate the embryo and support its development (Almiñana et al., 2012). From the mentioned preliminary studies, it is probable that the tubal fluid plays not only an important role in the survival of the sperm and embryo, but also regulates the immune reactions inside the lumen of the uterine tube. This is also why further research will be needed to accurately describe the mechanism of the relationship between tubal fluid and immune reactions inside the uterine tube.

## 11 Female sex hormone and the tubal immune privilege

Progesterone and estrogen are steroid female sex hormones produced cyclically by the ovaries. Within the female reproductive tract, the mucosal immune system is precisely regulated by both sex hormones to protect against potential pathogens without compromising fetal survival. This regulation leads to changes in innate and adaptive immune responses throughout the cycle and to differential regulation of the immune cell populations present in the female reproductive system. Sex hormones can act directly on cells or indirectly via intermediate cells to modulate cytokine and chemokine secretion (Wira et al., 2015). For example, both hormones are known to regulate the quantity of macrophages present within the uterus, as well as the expression of these macrophages in the anti-inflammatory, M2 polarization state (Chambers et al., 2021). For successful fertilization and embryo survival, the immune system must be modulated during mid-cycle of the menstrual cycle. On other hand, this period lasting 7–10 days, when components of innate, humoral, and cell-mediated immunity are suppressed by estrogen and/or progesterone, enhancing the potential for viral infection. The period of fertilization and early development of the embryo is also a period of vulnerability for HIV infection (Wira and Fahey, 2008).

Despite significant progress in reproductive immunology and endocrinology, much remains to be done to more fully understand the complexities of the immune system in the female reproductive system. According to Wira et al. (2015), the most important questions we need to get answers to include:

1) defining the balance between direct effects of sex hormones and their indirect effects that are mediated through cytokines, chemokines, and growth factors made by immune cells, stromal cells, and epithelial cells that modulate female reproductive tract mucosal immune function;
2) defining the role of the female reproductive tract tissue microenvironment in regulating immune phenotype and function given that blood immune cells are different from their counterparts in the female reproductive system;
3) determining the extent to which immune cells function vary with their site of residence in the female reproductive system (e.g., uterus vs. uterine tube).

Additionally, very little is known about the effect of endocrine disruptors on the function of immune cells within the female reproductive system. There have been numerous studies and reviews demonstrating effects of endocrine disruptors on embryonic development and germ cell development and these are discussed in detail elsewhere, even less is known about their effects on the immune system of the female reproductive system (Dunbar et al., 2012).

## 12 Conclusion and further perspectives

The human uterine tube has a diverse population of immunologically active cells representing both the innate and adaptive immune systems; including lymphocytes, macrophages, NK cells, dendritic cells, and mast cells. In comparison with the uterus and its endometrium, studies examining immune cell populations and their functions in the uterine tube are not as numerous. Recent years have revealed that the obsolete notion of uterine tubes as simply a passive tubular organ for gametes and embryos could not be further from the truth. Active cross-talk between spermatozoa, oocytes, preimplantation embryos, and the wall of the uterine tube (including immunocompetent cells) occurs before, at, as well as after fertilization; yet also during transport of the early embryo toward the uterine cavity. However, immune crosstalk between the pre-hatching embryo in uterine tube and the mother has received little attention (Talukder et al., 2020). Recent animal studies indicate that the pre-hatching bovine embryo secretes bioactive molecules that can be detected by epithelial and immune cells of the female reproductive system and induces anti-inflammatory effect (Talukder et al., 2017; Talukder et al., 2018). Therefore, understanding these processes and its translation into clinical practice could substantially impact further development of the field of reproductive medicine (improvement of fertility and pregnancy outcomes) and in the management of sexually transmitted infections. Additionally, understanding how immune protection changes in the female reproductive tract during the menstrual cycle or in menopause is essential to understanding the pathogenesis of gynecological cancers.

This review has some limitations due to a relative lack of data regarding immune cells in the wall of the human uterine tube. In various scientific reports there are inconsistent data in terms of the immune cell populations in the female reproductive tract; this discrepancy comes from differences in the sampling phase in the menstrual cycle, laboratory methods (histology *versus* flow cytometry), and antibodies used to identify immune cells. In conclusion, scientific research of the immune system in the female reproductive tract can help solve the puzzle of semi-allograft embryo/fetus acceptance during pregnancy and contribute to improved women’s health.
